# Genotyping for HLA risk alleles versus patch tests to diagnose anti-seizure medication induced cutaneous adverse drug reactions

**DOI:** 10.3389/fphar.2022.1061419

**Published:** 2022-11-21

**Authors:** Lisanne E. N. Manson, Patricia C. Y. Chan, Stefan Böhringer, Henk-Jan Guchelaar

**Affiliations:** ^1^ Department of Clinical Pharmacy and Toxicology, Leiden University Medical Center, Leiden, Netherlands; ^2^ Department of Biomedical Data Sciences, Leiden University Medical Center, Leiden, Netherlands

**Keywords:** carbamazepine, lamotrigine, oxcarbazepine, phenytoin, human leukocyte antigen, patch test, stevens-johnson syndrome, toxic epidermal necrolysis

## Abstract

**Aim:** To provide a comparison of genotyping for HLA risk alleles versus patch testing to determine which of these two tests is a better diagnostic tool for cutaneous hypersensitivity reactions caused by anti-seizure medication.

**Methods:** A literature study was performed in PubMed to assess the sensitivity and specificity of HLA genotyping and patch tests for identifying anti-seizure medication induced cutaneous hypersensitivity reactions.

**Results:** This study shows that HLA-B*15:02 genotyping shows high sensitivity for carbamazepine-induced SJS/TEN, especially in Han Chinese and Southeast Asian patients (66.7–100.0%) whereas the sensitivity of patch tests (0.0–62,5%), HLA-A*31:01 (0–50%) and HLA-B*15:11 (18.2–42.9%) are lower. On the contrary, for carbamazepine and phenytoin induced DRESS, patch tests (respectively 70.0–88.9% and 14.3–70.0%) show higher sensitivity than HLA tests (0–66.7% and 0–12.7%). Also for lamotrigine-induced DRESS patch tests perform better than HLA-B*15:02 (33.3–40.0 versus 0%). For anti-seizure medication induced MPE and for oxcarbazepine-induced SCARs more studies are needed.

**Conclusion:** Use of HLA-B genotyping may aid clinicians in the diagnosis of carbamazepine, phenytoin, lamotrigine and oxcarbazepine induced SJS/TEN, particularly in Han Chinese and Southeast Asian patients. On the other hand, patch tests seem to perform better in the diagnosis of carbamazepine and phenytoin induced DRESS.

## Introduction

Stevens-Johnson syndrome (SJS), toxic epidermal necrolysis (TEN) and drug reactions with eosinophilia and systemic symptoms (DRESS) are cutaneous adverse drug reactions (cADRs) characterized by epidermal necrosis and mucous membrane damage (Mockenhaupt, 2011). These severe cutaneous adverse reactions (SCARs) are rare but potentially fatal. Due to their high risk of mortality and high hospitalization rates, SCARs are considered a public health concern (Harr and French, 2010). Therefore, early diagnosis of drug-induced SCARs is crucial to reduce their clinical and financial burden. Anti-seizure medication (ASM), particularly those containing aromatic rings, such as carbamazepine, lamotrigine, oxcarbazepine and phenytoin are a major cause of SCARs (Yang et al., 2011; Ordonez et al., 2015).

Currently, patch tests are used to identify culprit drugs after rash development. These tests can reproduce drug-induced delayed hypersensitivity reactions and have a low risk of systemic reactions due to moderate drug re-exposure of patients (Romano et al., 2008; Barbaud, 2014). However, there is a possibility that reactivation of SCARs will occur during patch testing (Wolkenstein et al., 1996). Therefore, it is important to evaluate the risk-benefit ratio before patch testing is performed. Although patch tests are easy to perform and relatively safe in milder cADRs such as maculopapular exanthema (MPE), these tests still encounter some disadvantages. For instance, interpretation of patch test results is complex and requires sufficient skill and knowledge (Lazzarini et al., 2013). Moreover, it takes approximately five to 7 days to complete a patch test, which is inconvenient for patients (Davis et al., 2008). Furthermore, the diagnostic value of these tests in the diagnosis of SCARs is still largely unknown. Therefore, further research is needed to determine the diagnostic test criteria of patch testing.

Although early diagnosis of SCARs is still not optimal, advances in the pharmacogenomics of SCARs have led to the discovery of human leukocyte antigen (HLA) associations with drug-induced SCARs (Collins et al., 2016). Testing for these so-called HLA risk alleles is a potential method for identifying patients at risk for SCARs. Important to note is that the strength of the associations between HLA risk alleles and SCARs differs between ethnicities, presumably because of the differences in prevalence of the HLA risk alleles between ethnic groups (Alfirevic and Pirmohamed, 2011). Previous findings suggest that HLA-B*15:02 is strongly associated with carbamazepine-induced SJS/TEN in the Han Chinese population in Taiwan, mainland China and Hong Kong (Chung et al., 2004; Man et al., 2007; Wang et al., 2011; Shi et al., 2012). The same drug-gene interaction is also reported in Southeast Asian populations, such as Malay, Thai and Vietnamese (Tassaneeyakul et al., 2010; Chang et al., 2011; Kulkantrakorn et al., 2012; Nguyen et al., 2015). HLA-B*15:02 is also strongly associated with phenytoin-induced SJS and TEN (Li et al., 2015). Lamotrigine and oxcarbazepine users have a lower risk than carbamazepine and phenytoin of developing SCARs but there is also evidence of interactions between lamotrigine and oxcarbazepine with HLA-B*15:02 (Li et al., 2015; Tangamornsuksan et al., 2018).

Whereas in most Asian populations ASM-induced SCARs are associated with HLA-B*15:02, in Caucasian and Korean populations, ASM-induced SCARs, particularly DRESS, are associated with HLA-A*31:01 (McCormack et al., 2011; Ozeki et al., 2011; Yip and Pirmohamed, 2017). In Japanese patients, ASM-induced SCARs are associated with HLA-B*15:11 (Kaniwa et al., 2010).

HLA testing in certain Southeast Asian populations is recommended according to the Clinical Pharmacogenetics Implementation Consortium (CPIC) guidelines (Phillips et al., 2018; Karnes et al., 2021). Besides, the Dutch Pharmacogenetics Working Group (DPWG) guidelines advise pharmacists and other healthcare providers to dispense or prescribe alternative treatments to new carbamazepine-users carrying an HLA-B*15:02 allele, when possible. Both the CPIC and the DPWG guidelines recommend avoiding not only carbamazepine, but also phenytoin and oxcarbazepine in HLA-B*15:02 positive patients, while DPWG also recommends avoiding lamotrigine if an alternative is available (PharmGKB, 2021a; PharmGKB, 2021c; PharmGKB, 2021d; PharmGKB, 2021b). Compared to HLA-B*15:02 carriers, HLA-A*31:01 and HLA-B*15:11 positive patients have a lower risk of developing ASM-induced SCARs. In these patients, an alternative treatment needs to be considered, if the risks outweigh the benefits (PharmGKB, 2021a). The HLA genotyping tests may also be used to ascertain the diagnosis of ASM-induced cutaneous hypersensitivity reactions as an alternative for patch tests.

Previous studies have only focused on assessing the diagnostic test criteria of either patch tests or HLA tests. Besides, studies on HLA genotyping and drug hypersensitivity reactions focused mainly on the use of HLA genotyping in a preventive setting instead of using it as a tool for diagnosis. HLA testing is not implemented in the clinics in a diagnostic setting, after a cADR has already occurred, possibly due to a lack of knowledge on their diagnostic criteria compared to patch tests (Lin et al., 2013). Thus, comparison of the sensitivity and specificity of HLA risk allele testing versus patch testing in diagnosing cADRs is needed to improve the diagnosis of potentially life-threatening ASM-induced SCARs. This literature study compares the cADRs diagnostic test criteria between HLA tests and patch tests in terms of sensitivity and specificity in order to improve the diagnosis of cADRs.

## Methods

### Search strategy

A literature search using the PubMed database was conducted on 30 April 2021. The search was performed using the following MeSH and/or search terms: HLA, drug hypersensitivity, anticonvulsant, carbamazepine, oxcarbazepine, lamotrigine, phenytoin, patch test and synonyms. The title and abstract of all records were screened. Furthermore, reference lists from the included papers were manually checked to identify additional relevant studies.

### Study selection and data extraction

Case-control and cohort studies were included with controls and cases using ASMs with the outcomes SJS/TEN, DRESS and MPE. Furthermore, only studies concerning patch tests or HLA-A*31:01, HLA-B*15:02 or HLA-B*15:11 genotyping in relation to carbamazepine, lamotrigine, oxcarbazepine or phenytoin-induced cADRs were included. Duplicates, case reports, reviews and non-English articles were excluded.

The papers were then categorized and presented in tables per drug and cADR. In addition, the numbers of true positive, true negative, false positive and false negative participants were used to calculate the sensitivities and specificities of each test. Sensitivity is the percentage of subjects with an cADR who test positive for the HLA risk allele while specificity is the percentage of subjects without an cADR who test negative for the HLA risk allele. The sensitivity and specificity were calculated as explained in the article by Tonk et al. (Tonk et al., 2017). In the tables the HLA carrier frequencies are shown derived from the Allele frequency net database (Gonzalez-Galarza et al., 2020). Comparisons between patch tests and HLA tests were made if studies in populations with similar ethnicities were found. The sample size of the studies, including the number of cases and controls used, can be found in Supplementary Table S1.

## Results

### Study selection

**FIGURE 1:**
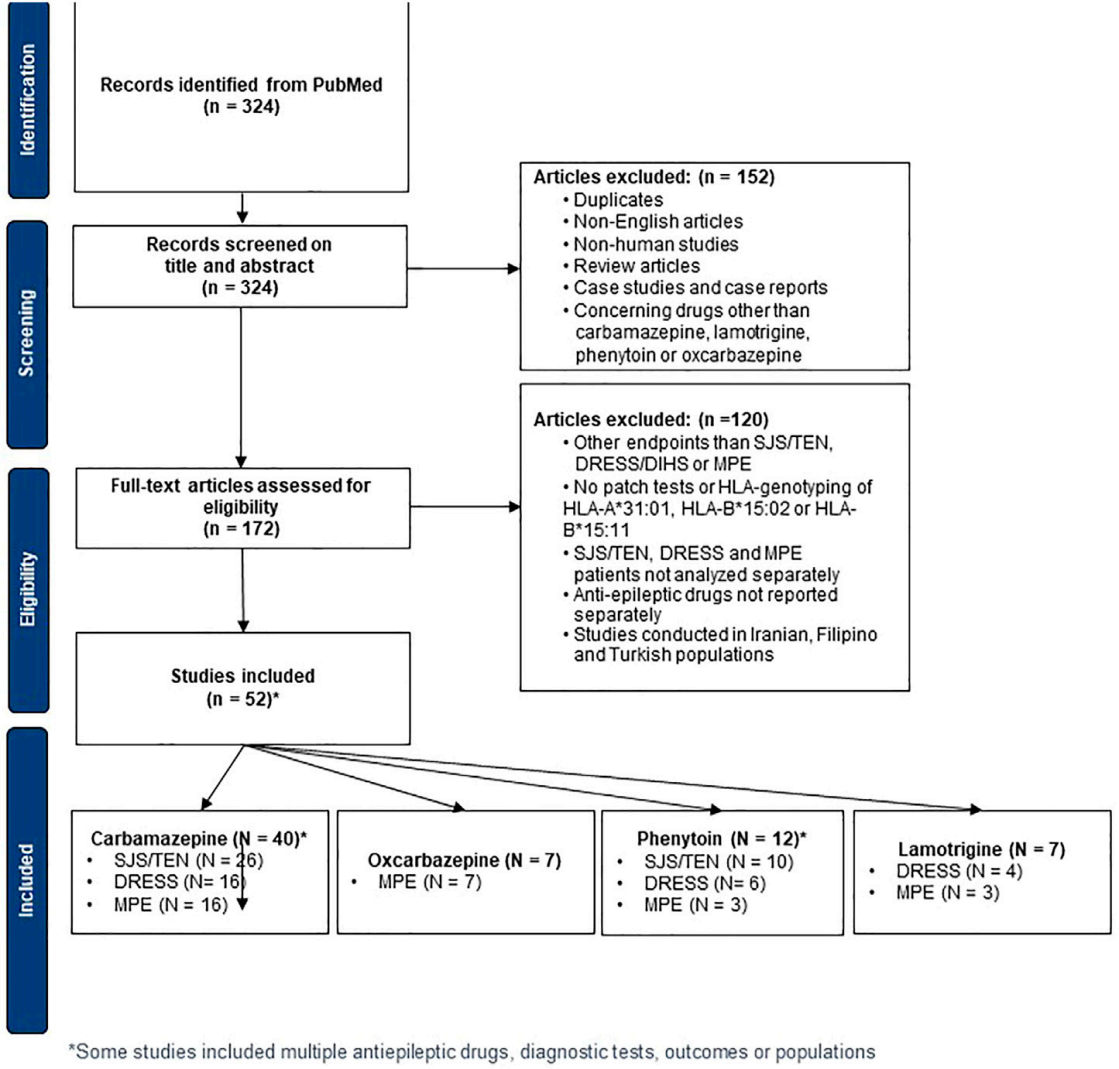
Flow Chart of study selection according to inclusion and exclusion criteria.

The literature search yielded 324 records which were screened on title and abstract, followed by assessing the full-text articles for eligibility. This resulted in the inclusion of 55 relevant case-control and cohort studies in total. These studies contained data to calculate the sensitivity and specificity of patch tests, HLA-B*15:02 tests, HLA-B*15:11 tests and/or HLA-A*31:01 tests. The study selection procedure is visualized in Figure 1.

### Carbamazepine

The sensitivity and specificity of patch tests and HLA-B*15:02, HLA-B*15:11 and HLA-A*31:01 for confirming carbamazepine induced SJS/TEN are displayed in Table 1. All HLA tests show high specificity (76.0–100.0%) as do patch tests (100.0%). As was also seen in our previous study (Manson et al., 2020), HLA-B*15:02 has high sensitivity for confirming carbamazepine-induced SJS/TEN in Han Chinese, Taiwanese and Southeast Asian populations (66.7–100%) but low sensitivity in Caucasians and Koreans (0.0–50.0%) HLA-B*15:11 has a much lower sensitivity for confirming carbamazepine-induced SJS-TEN with a sensitivity of only 18.2–25.0% in Japanese and Koreans. HLA-A*31:01 has low sensitivity for confirming carbamazepine induced SJS-TEN (0.0–50.0%), especially in Han Chinese and Malay (0.0–1.9%). Just like the HLA-B*15:02 test, patch tests have low sensitivity for carbamazepine-induced SJS/TEN in Caucasians (0.0–14.3%) but high sensitivity in Taiwanese patients (62.5%). However, this sensitivity was still lower than that of the HLA-B*15:02 test in Han Chinese and Taiwanese patients (77.4–100.0%).

**TABLE 1:** Sensitivity and specificity of patch tests and HLA tests for carbamazepine-induced SJS/TEN.

Test	Population	Sensitivity	Specificity	References	Carrier frequency (%)
Patch test	Taiwanese	62.5%	100.0%	Lin et al. (2013)	
	Caucasian	0.0–14.3%	100.0%	(Wolkenstein et al., 1996; Barbaud et al., 2013)	
HLA-B*15:02	Han Chinese/Taiwanese	77.4–100.0%	88.1–97.0%	Chung et al. (2004), Hung et al. (2006), Cheung et al. (2013), Lin et al. (2013), Genin et al. (2014), Hsiao et al. (2014)	0–59
	Southeast Asian^a^	66.7–100.0%	76.0–100.0%	Tassaneeyakul et al. (2010), Kulkantrakorn et al. (2012), Nguyen et al. (2015), Locharernkul et al. (2008), Then et al. (2011), Toh et al. (2014), Khor et al. (2017), Yuliwulandari et al. (2017), Sukasem et al. (2018), Huyen et al. (2020)	5–35
	Indian	22.2–96.4%	96.0–100.0%	Aggarwal et al. (2014), Khor et al. (2014), Khor et al. (2017), Devi, (2018)	0–12
	Caucasian	0.0–50.0%	100.0%	Genin et al. (2014), Ramirez et al. (2017)	0–1.2
	Korean	14.3%	100.0%	Kim et al. (2011)	0.4–4.4
HLA-B*15:11	Japanese	18.2%	99.0%	Kaniwa et al. (2010)	0.8–1.8
	Korean	25.0%	96.0%	Kim et al. (2011)	3.3–4.0
HLA-A*31:01	Han Chinese	0.0–1.9%	95.8–97.2%	Hung et al. (2006), Genin et al. (2014), Hsiao et al. (2014), Khor et al. (2017)	0.8–12.6
	Malay	0.0%	93.8%	Khor et al. (2017)	0.8–4.1
	Indian	0.0–50.0%	91.2–95.7%	Khor et al. (2017), Ihtisham et al. (2019)	6.9
	Caucasian	0.0–41.7%	96.1–100.0%	McCormack et al. (2011), Genin et al. (2014), Ramirez et al. (2017)	3.0–13.4
	Korean	42.9%	86.0%	Kim et al. (2011)	11.2

^a^ Includes Indonesian, Malay, Thai and Vietnamese populations due to comparable HLA allele frequencies.

For the diagnosis of carbamazepine-induced DRESS, HLA-B*15:02 has a sensitivity of 0.0% in most populations except for South-East Asian populations (20.0–66.7%) (Table 2). Also HLA-B*15:11 shows a sensitivity of 0% in Koreans. On the other hand, the HLA-A*31:01 test has relatively high sensitivity in Caucasians and Koreans (34.6–70.0%). Patch tests have even higher sensitivity for diagnosing carbamazepine-induced DRESS in Taiwanese, Indian, Caucasian and Japanese (70.0–88.9) while the specificity also remains high with 100.0%. Patch tests therefore seem to perform better in diagnosing carbamazepine-induced DRESS than HLA tests.

**TABLE 2:** Sensitivity and specificity of patch tests and HLA tests for carbamazepine-induced DRESS.

Test	Population	Sensitivity	Specificity	References	Carrier frequency (%)
Patch test	Taiwanese	70.0%	100.0%	Lin et al. (2013)	
	Indian	83.0%	-	Shiny et al. (2017)	
	Caucasian	76.5–84.6%	100.0%	Santiago et al. (2010), Barbaud et al. (2013)	
	Japanese	88.9%	-	Lammintausta and Kortekangas-Savolainen, (2005)	
HLA-B*15:02	Han Chinese/Taiwanese	0.0%	90.0–95.8%	Hung et al. (2006), Lin et al. (2013), Genin et al. (2014), Hsiao et al. (2014)	0–59
	Southeast Asian^a^	20.0–66.7%	76.0–95.9%	Nguyen et al. (2015), Sukasem et al. (2018)	5–35
	Caucasian	0.0%	100.0%	Genin et al. (2014), Ramirez et al. (2017)	0–1.2
	Korean	0.0%	100.0%	Kim et al. (2011)	0.4–4.4
HLA-B*15:11	Korean	0.0%	96.0%	Kim et al. (2011)	3.3–4.0
HLA-A*31:01	Han Chinese	15.4–50.0%	95.8–97.2%	Hung et al. (2006), Genin et al. (2014), Hsiao et al. (2014)	0.8–12.6
	Indian	0.0%	95.7%	Ihtisham et al. (2019)	6.9
	Caucasian	34.6–70.0%	95.7–97.5%	McCormack et al. (2011), Genin et al. (2014), Ramirez et al. (2017)	3.0–13.4
	Korean	58.8%	86.0%	Kim et al. (2011)	11.2

^a^ Includes Indonesian, Malay, Thai and Vietnamese populations due to comparable HLA allele frequencies.

For carbamazepine-induced MPE, results can be seen in Table 3. HLA-B*15:02 and HLA-A*31:01 have low sensitivity for carbamazepine-induced MPE, respectively 5.6–23.5% and 13.7–50.0%. Patch tests in Korean and Japanese have a higher sensitivity of 61.5–66.7% with a specificity of 100%. Studies concerning patch tests for carbamazepine-induced MPE in Caucasians show high variability in sensitivity which ranges from 18.9 to 85.7% while the specificity in all studies is high (90.9–100.0%).

**TABLE 3:** Sensitivity and specificity of patch tests and HLA tests for carbamazepine-induced MPE.

Test	Population	Sensitivity	Specificity	References	Carrier frequency (%)
Patch test	Caucasian	18.9–85.7%	90.9–100.0%	Houwerzijl et al. (1977), Motley and Reynolds, (1989), Alanko, (1993), Jones et al. (1994), Troost et al. (1996), Lammintausta and Kortekangas-Savolainen, (2005), Romano et al. (2006)	
	Korean	61.5%	100.0%	Lee et al. (2003)	
	Japanese	66.7%	-	Osawa et al. (1990)	
HLA-B*15:02	Han Chinese	5.6–7.8%	92.8–95.8%	Hung et al. (2006), Hsiao et al. (2014)	0–59
	Thai	22.2–23.5%	81.0–95.9%	Locharernkul et al. (2008), Sukasem et al. (2018)	16
HLA-A*31:01	Han Chinese	13.7–33.3%	96.7–97.2%	Hung et al. (2006), Hsiao et al. (2014)	0.8–12.6
	Indian	22.2%	95.7%	Ihtisham et al. (2019)	6.9
	Caucasian	21.7–50.0%	96.1–100.0%	McCormack et al. (2011)	3.0–13.4

### Oxcarbazepine

For oxcarbazepine-induced hypersensitivity, studies on patch tests are scarce. Results are shown in Table 4. Only studies on oxcarbazepine-induced MPE in Caucasian populations were found. The patch test has a very low sensitivity (12.5%–13.6%) but high specificity for oxcarbazepine-induced MPE. Also for the HLA-B*15:02 test the sensitivity is low; 3.6–44.4% in Han Chinese and 0.0% in Korean. Since the studies in patch tests were performed in Caucasian populations and the studies on HLA-B-15:02 in Asian populations, a comparison between the tests cannot be made.

**TABLE 4:** Sensitivity and specificity of patch tests and HLA tests for oxcarbazepine-induced MPE.

Test	Population	Sensitivity	Specificity	References	Carrier frequency (%)
Patch test	Caucasian	12.5–13.6%	100.0%	Troost et al. (1996), Lammintausta and Kortekangas-Savolainen, (2005)	
HLA-B*15:02	Han Chinese	2.9–44.4%	88.9–94.3%	Hu et al. (2011), He et al. (2012), Lv et al. (2013), Chen et al. (2017)	0–59
	Korean	0.0%	98.6%	Moon et al. (2016)	0.4–4.4

### Phenytoin

As can be seen in Table 5, only one study concerning patch tests and phenytoin-induced SJS/TEN was found. In an Indian population, patch tests have a sensitivity of 0%. Similarly, the HLA-B*15:02 test has a sensitivity of 0% in two Indian populations for phenytoin-induced SJS/TEN. Also in a Caucasian population the HLA-B*15:02 has a sensitivity of 0% while in Han Chinese, Taiwanese and Southeast Asian populations, the sensitivity is higher (12.8–100%). Because of the lack of studies concerning patch tests in these populations, no comparison could be made on the patch test versus the HLA-B*15:02 test.

**TABLE 5:** Sensitivity and specificity of patch tests and HLA tests for phenytoin-induced SJS/TEN.

Test	Population	Sensitivity	Specificity	References	Carrier frequency (%)
Patch test	Indian	0.0%	-	Shiny et al. (2017)	
HLA-B*15:02	Han Chinese/Taiwanese	30.8–46.7%	80.0–94.9%	Hung et al. (2010), Cheung et al. (2013), Su et al. (2019)	0–59
	Southeast Asian^a^	12.8–100.0%	78.1–85.9%	Locharernkul et al. (2008), Tassaneeyakul et al. (2016), Chang et al. (2017)	5–35
	Indian	0.0%	100.0%	Aggarwal et al. (2014), Devi, (2018)	0–12
	Caucasian	0.0%	100.0%	Ramirez et al. (2017)	0–1.2

^a^ Includes Indonesian, Malay, Thai and Vietnamese populations due to comparable HLA allele frequencies.

For phenytoin-induced DRESS (Table 6), the HLA-B*15:02 test has low sensitivity in both Asian (0.0–12.7%) and a Caucasian population (0.0%). Only 2 studies were found on patch tests. The sensitivity of patch tests in these studies is higher than for HLA-B*15:02. In a Caucasian population a sensitivity of 14.3% was seen with a specificity of 100% while the sensitivity was 70% in an Indian population.

**TABLE 6:** Sensitivity and specificity of patch tests and HLA tests for phenytoin-induced DRESS.

Test	Population	Sensitivity	Specificity	References	Carrier frequency (%)
Patch test	Indian	70.0%	—	Shiny et al. (2017)	
	Caucasian	14.3%	100.0%	Santiago et al. (2010)	
HLA-B*15:02	Taiwanese	12.7%	94.9%	Su et al. (2019)	8.4–12
	Southeast Asian^a^	0.0–9.5%	78.1–85.9%	Tassaneeyakul et al. (2016), Chang et al. (2017)	5–35
	Caucasian	0.0%	100.0%	Ramirez et al. (2017)	0–1.2

^a^ Includes Indonesian, Malay, Thai and Vietnamese populations due to comparable HLA allele frequencies.

For phenytoin-induced MPE, the studies were limited (Table 7). Only one study on patch tests was found in a Finnish population where the sensitivity was 33.3%. The sensitivity of HLA-B*15:02 in Taiwanese and Thai populations is a little bit lower: 11.2–25%. Due to the differences in ethnicities between the available studies, a comparison cannot be made.

**TABLE 7:** Sensitivity and specificity of patch tests and HLA tests for phenytoin-induced MPE.

Test	Population	Sensitivity (%)	Specificity (%)	References	Carrier frequency (%)
Patch test	Caucasian	33.3	100.0	Lammintausta and Kortekangas-Savolainen, (2005)	
HLA-B*15:02	Taiwanese	11.2	94.9	Su et al. (2019)	8.4–12
	Thai	25.0	82.2	Locharernkul et al. (2008)	16

### Lamotrigine

For lamotrigine, only patch tests on lamotrigine-induced DRESS (Table 8) and MPE (Table 9) were found. HLA-B*15:02 has a sensitivity of 0.0% for lamotrigine-induced DRESS in Caucasians with a specificity of 100%. Also a patch test has a specificity of 100% but it has a sensitivity of 33.3–40.0% in Caucasians which is considerably higher than the sensitivity of HLA-B*15:02. On lamotrigine-induced MPE only one study researching patch tests and two on HLA-B*15:02 were found. The sensitivity of a patch test was 0% in a Caucasian population. Also the sensitivity of HLA-B*15:02 in Mexican Mestizo was 0% while it was much higher (50.0%) in a Thai population.

**TABLE 8:** Sensitivity and specificity of patch tests and HLA tests for lamotrigine-induced DRESS.

Test	Population	Sensitivity	Specificity (%)	References	Carrier frequency
Patch test	Caucasian	33.3–40.0%	100.0	Galindo et al. (2002), Santiago et al. (2010)	
HLA-B*15:02	Caucasian	0.0%	100.0	Kazeem et al. (2009), Ramirez et al. (2017)	0–1.2
	Han Chinese	33.3%	86.7	Cheung et al. (2013)	0–59
	Thai	0.0%	88.08	Koomdee et al. (2017)	16

**TABLE 9:** Sensitivity and specificity of patch tests and HLA tests for lamotrigine-induced MPE.

Test	Population	Sensitivity (%)	Specificity (%)	References	Carrier frequency (%)
Patch test	Caucasian	0.0	100.0	Lammintausta and Kortekangas-Savolainen (2005)	
HLA-B*15:02	Thai	50.0	88.0	Koomdee et al. (2017)	16
	Mexican Mestizo	0.0	100.0	Fricke-Galindo et al. (2014)	0.0–3.0

## Discussion

The aim of this literature study was to determine whether patch tests or HLA risk allele genotyping perform better in diagnosing ASM-induced cutaneous hypersensitivity reactions. Such testing occurs in a retrospective setting: After the hypersensitivity occurred, drug-associations needs to be established to prevent future exposure. As such, the nature of testing is not predictive. Rather, the question is whether the presence of a known risk factor can be established. This naturally leads to assessing the performance of the HLA and patch tests by using the sensitivity and specificity, as sensitivity measures the percentage of patients for which the risk factor can be detected and specificity how often that factor can be excluded. Both measures can be evaluated on case-control data. Sensitivity and specificity cannot be evaluated independently, as for continuous thresholds, sensitivity increases when specificity decreases and *vice versa*. In our study, the predictor is binary (present or not) leading to fixed values of sensitivity and specificity. Specificity is higher than 90% for both patch- and HLA-testing, although specificity seems to be somewhat higher for patch testing. This might be of importance, when avoidance of false positives is of importance.

In this study, we have examined the cutaneous hypersensitivity reactions SJS/TEN, DRESS and MPE with regard to the drugs carbamazepine, lamotrigine, oxcarbazepine and phenytoin as described in the CPIC and DWPG guidelines. A main finding of this study is that HLA-B*15:02 tests are more sensitive than HLA-B*15:11, HLA-A*31:01 and patch tests for detecting carbamazepine-induced SJS/TEN in Han Chinese and Southeast Asian populations. However, patch testing is most sensitive and specific for the diagnosis of carbamazepine, phenytoin and lamotrigine induced DRESS. For diagnosis of ASM-induced MPE, sensitivities of both HLA and patch tests are low.

To our knowledge, this is the first study that determined whether HLA genotyping or patch testing is better in diagnosing ASM-induced SCARs. Although previous studies have already examined the diagnostic utility of patch tests or HLA risk allele tests for identifying SCARS, most of these studies evaluated the diagnostic test criteria of HLA genotyping for pre-emptive use to predict ASM-induced SCARs, whereas this study focuses on the use of HLA genotyping as a diagnostic tool for identifying ASM-related SCARs. Because the number of studies on the diagnostic utility of patch tests and HLA tests for identifying SCARs are limited, more insight into the diagnostic test criteria is needed before HLA testing could be implemented for patients presenting with cutaneous hypersensitivity reactions. Our study provides a clear overview of the available data on the sensitivity and specificity of patch tests and HLA tests in order make a comparison of these tests for identifying SCARs. This may contribute to improvement of the diagnosis of SCARs.

The endpoints used in this study were based on the endpoints mentioned in the DPWG and CPIC guidelines concerning the ASMs (Koninklijke Nederlandse Maatschappij ter bevordering der Pharmacie, 2016; Koninklijke Nederlandse Maatschappij ter bevordering der Pharmacie, 2018a; Koninklijke Nederlandse Maatschappij ter bevordering der Pharmacie, 2018b; Koninklijke Nederlandse Maatschappij ter bevordering der Pharmacie, 2018c; Phillips et al., 2018; Karnes et al., 2021). We chose to only include studies that classified hypersensitivity reactions because the sensitivity and specificity of these two diagnostic tests vary depending on the condition. In general, the sensitivity of HLA genotyping is the highest when identifying SJS/TEN followed by DRESS and then MPE. Therefore, studies that combined SCARs as one endpoint were excluded from analysis to obtain the most accurate results.

A limitation of this study is that publication bias may be present as most papers at least show one statistically significant association. This fact could potentially lead to an overestimation of sensitivity and specificity for the diagnostic tests which may not fully translate to patients in real-world practice, although the strong associations reported here are not likely to exhibit strong bias. For example with oxcarbazepine-induced SJS/TEN, only one study found a strong association with HLA-B*15:02 and high sensitivities and specificities for identifying SJS/TEN (Chen et al., 2017). Moreover, there is limited evidence of the utility of HLA risk allele testing in populations other than Han Chinese and Southeast Asian populations, especially for identifying lamotrigine and oxcarbazepine-induced SCARs. Another limitation is that there is limited data available for patch testing and some HLA-drug interactions due to the rarity of SCARs. Besides, the majority of the included studies has a small sample size. Because of the limited number of studies, no minimum sample size was used. All studies were used. The results from the smaller studies may be less reliable than the results from studies with a larger number of cases and controls but they might have a big impact on the results shown in the tables. For instance for carbamazepine-induced SJS/TEN, the sensitivity of HLA-B*15:02 was 0.0–50% in Caucasians and Koreans. However, the sensitivity of 50% was found in a study containing only 2 cases. This was the same study that found a low sensitivity of HLA-A*31:01 of 0% in Caucasians. In order to increase the sample size, there is need for larger multicenter studies. For patch testing, there were only few studies that mainly assessed the diagnostic test criteria of patch testing with carbamazepine in Caucasian subjects. Since the sensitivity and specificity differ importantly between populations, comparisons between different ethnicities are unreliable. Therefore, in a future study it would also be interesting to examine whether ethnicity influences the sensitivity and specificity of patch tests. The limited availability of patch test studies on diagnosing SCARs is possibly due to the risk of inducing a hypersensitivity reaction.

Due to the limited articles available on patch tests and HLA tests, all available studies were taken into account, regardless the sample size, differences in patient selection and definition and diagnostic confirmation of cADRs. Most of the studies did use ASM-tolerant subjects as controls. But there were differences in patient selection and definition of the cADRs, making a direct comparison between patch tests and HLA tests difficult and not very precise. However, because of the limited data available, using all available studies was the best that could be done.

Patch test studies for identifying ASM-induced hypersensitivity reactions, and studies of HLA genotyping to identify lamotrigine and oxcarbazepine-related SCARs are limited. Even though the evidence is limited, the use of patch tests and HLA genotyping seems promising for identifying ASM-induced SCARs, specifically DRESS. However, more and larger studies on the diagnostic test criteria are needed to determine the sensitivity and specificity more accurately, and confirm the effectiveness of both patch testing and HLA genotyping. Overall, our study provides an overview of the diagnostic criteria of patch tests and HLA risk allele tests for identifying ASM-induced SCARs and it contributes to a better understanding of the utility of these tests as a diagnostic tool. However, we believe that the limitations do not affect the outcome of this study and the results contribute to a better understanding of the diagnostic test criteria of HLA genotyping and patch testing.

To conclude, this study compared the diagnostic test criteria of patch tests and HLA genotyping to assess whether patch testing or HLA genotyping performs better in diagnosing ASM-induced cutaneous hypersensitivity reactions. The sensitivity and specificity of HLA-B*15:02 are high for SJS/TEN caused by carbamazepine in Southeast Asian and Han Chinese patients. HLA-B*15:02 genotyping for identifying SJS/TEN shows a higher sensitivity and specificity, especially in Southeast Asians and Han Chinese than patch testing, and is also safer than patch tests. Thus, implementation of HLA-B*15:02 genotyping as a diagnostic tool to identify SJS/TEN should be considered. For the diagnosis of carbamazepine, lamotrigine and phenytoin induced DRESS, patch testing shows a higher sensitivity than HLA genotyping and a specificity of nearly 100%, suggesting that patch tests could be used to rule out if the hypersensitivity reaction is ASM-related. Implementing HLA-B*15:02 testing may aid clinicians in the diagnosis of ASM-induced SJS/TEN. This may result in correctly, safer and faster identification of SCARs.

## Data Availability

The original contributions presented in the study are included in the article/Supplementary Material, further inquiries can be directed to the corresponding author.
